# Correlation of biochemical and imaging markers with hepatic adenoma in patients with glycogen storage disease: a retrospective single-center study

**DOI:** 10.1186/s13023-026-04239-z

**Published:** 2026-02-06

**Authors:** Jhii-Hyun Ahn, Seung Whan Cha, Yunkoo Kang

**Affiliations:** 1 Department of Radiology, Wonju Severance Christian Hospital, Yonsei University Wonju College of Medicine, Wonju, Republic of Korea; 2 Department of Pediatrics, Wonju Severance Christian Hospital, Yonsei University Wonju College of Medicine, Wonju, Republic of Korea

**Keywords:** FibroScan, Gamma-glutamyl transferase, Glycogen storage disease, Hepatic adenoma, Shear wave elastography, Triglycerides

## Abstract

**Background:**

Hepatic adenoma is a serious complication of glycogen storage disease, particularly types I and III. However, noninvasive predictors of adenoma presence and progression for use in clinical practice remain limited. In this retrospective study, we included 93 patients with genetically confirmed glycogen storage disease who underwent liver ultrasonography, FibroScan, shear wave elastography, and routine biochemical testing between December 2020 and March 2025. Patients with and without hepatic adenoma were compared to identify discriminative variables, which were used to construct a logistic regression model. Patients with serial imaging data were assessed for changes in adenoma size and clinical parameters.

**Results:**

Of the 93 patients included, 13 (14%) had hepatic adenomas. Age, gamma-glutamyl transferase levels, triglyceride levels, liver stiffness measured by FibroScan, and total cholesterol levels were significantly elevated in patients with adenoma compared to those without adenoma (*p* < 0.05). A logistic regression model combining age, gamma-glutamyl transferase, triglycerides, liver stiffness as measured by FibroScan, total cholesterol, and liver stiffness as measured by shear wave elastography achieved an area under the curve of 0.87. Adenoma progression was accompanied by changes in gamma-glutamyl transferase levels and the FibroScan Controlled Attenuation Parameter. The simplified thresholds of gamma-glutamyl transferase > 60 IU/L, triglycerides > 300 mg/dL, liver stiffness by FibroScan > 6.0, FibroScan Controlled Attenuation Parameter > 280 dB/m, and total cholesterol > 220 mg/dL were determined from group comparisons and the logistic regression model.

**Conclusions:**

The findings suggest that routine biochemical markers, together with selected elastographic parameters for supportive assessment, can aid in the detection and risk stratification of hepatic adenomas in patients with glycogen storage disease. The use of data-derived clinical thresholds may help guide surveillance strategies and facilitate earlier identification of patients potentially at increased risk.

## Background

Glycogen storage diseases (GSDs) are a group of rare inherited metabolic disorders characterized by the abnormal accumulation of glycogen in various tissues, most notably the liver [1]. Among the long-term hepatic complications associated with GSD, hepatic adenomas represent a particularly significant concern because of their potential for hemorrhage or malignant transformation [2, 3]. Patients with GSD type I have a lifetime hepatic adenoma risk of up to 70–80%, whereas GSD type III carries a risk of approximately 20–40%. Current European Study on Glycogen Storage Disease and American College of Medical Genetics and Genomics guidelines recommend annual ultrasonography with periodic magnetic resonance imaging, highlighting the need for improved early detection strategies [4, 5]. Identifying patients at a high risk of adenoma development remains challenging.

Although prior studies have explored genotype–phenotype correlations and identified individual risk factors, data on the ability of routine imaging and biochemical parameters, such as FibroScan, shear wave elastography (SWE), and liver function test results, to predict adenomas are lacking. Identifying noninvasive markers of adenomas could substantially improve long-term surveillance strategies in GSD management [6, 7]. 

Previous reports have primarily focused on long-term cumulative incidence or descriptive case series, often without standardized liver stiffness (LS) measurements or serial biochemical tracking [8, 9]. Whether routinely collected clinical data can offer early insights into adenoma risk therefore remains to be determined. Tools that can not only detect existing adenomas but also anticipate their progression over time are required.

In this single-center retrospective study, we aimed to evaluate the clinical, laboratory, and imaging factors associated with the presence and progression of hepatic adenoma in a Korean cohort of patients with GSD. Additionally, we determined clinical thresholds for statistically significant markers to guide real-world surveillance and intervention strategies. Despite longstanding recognition of metabolic imbalance as a major contributor to adenoma development, no previous study has integrated routine biochemical markers with elastography to construct a predictive model for hepatic adenoma in GSD. Our work therefore addresses an important unmet clinical need.

## Methods

### Study design and population

This retrospective observational study was conducted at Yonsei University Wonju College of Medicine. The medical records of 93 patients with genetically confirmed GSD, who had undergone a total of 317 rounds of liver ultrasound, SWE, FibroScan, and biochemical testing between December 2020 and March 2025, were reviewed. GSD subtype classification identified 56 patients with type Ia, 4 patients with type Ib, 10 patients with type III, 2 patients with type VI, 20 patients with type IX, and 1 patient with an unknown type. This study was approved by the Institutional Review Board of Yonsei University Wonju College of Medicine (No. CR325018).

### Data collection

Data including age, sex, GSD subtype, LS as measured by FibroScan and SWE, and laboratory test results indicating the levels of aspartate aminotransferase (AST), alanine aminotransferase (ALT), gamma-glutamyl transferase (GGT), total cholesterol, triglycerides, uric acid, and Mac-2 binding protein glycosylation isomer (M2BPGi) were extracted from the electronic medical records. These laboratory variables were selected a priori due to their metabolic relevance in GSD (hyperlipidemia, cholestasis, hepatic inflammation). M2BPGi was included as an exploratory fibrosis marker. Because fasting is unsafe in patients with GSD, routine biochemical testing was performed in the non-fasting state, with no changes made to the patient dietary regimen.

### Adenoma classification and dynamics

The presence of an adenoma was determined based on ultrasound findings. For the dynamic analysis, adenoma size change status (increase, decrease, or stability) was used as the primary classification criterion for Table 3. Changes in adenoma size were assessed at the lesion level using serial imaging data, with each adenoma compared to its size at the immediately preceding imaging visit. Adenomas were categorized as increased, decreased, or stable based on the direction of size change, and changes in laboratory and imaging parameters were correlated with these size-based categories.

### FibroScan

LS and fat content were evaluated in patients with GSD by performing transient elastography using a FibroScan device (Echosens, Paris, France). LS measurements were reported in kilopascals (kPa), while fat content was quantified using the Controlled Attenuation Parameter (CAP). For each patient, multiple LS measurements were obtained, and the median value of at least 10 valid measurements, each with an interquartile range of < 30% of the median, was used for analysis to ensure reliability and accuracy [10]. 

### Shear wave elastography

Two-dimensional SWE was conducted to evaluate LS using a convex i8CX1 probe (Aplio i800, Canon Medical Systems Corp., Otawara, Japan). The system displays a color-coded map of the spatial distribution of elasticity values (acquisition box) superimposed on a B-mode image. It also displays a propagation map that reveals shear-wave arrival time contours. The two maps were viewed simultaneously, with the propagation map used for quality control. The manufacturer’s measurement protocol recommends placing a 7 mm diameter region of interest in the area with the most parallel propagation contours, preferably in the upper part of the acquisition box. For each examination, between three and five regions of interest were placed in the right liver, avoiding vascular and biliary structures. The final LS value was expressed in kPa as the average of at least three valid measurements.

### Statistical analysis

Descriptive statistics were calculated for the entire cohort. Patients with and without adenomas were compared using the Mann–Whitney U test for continuous variables. Variables associated with the presence of adenoma were identified using logistic regression, and predictive performance was assessed using receiver operating characteristic curves. All statistical analyses were performed using Python 3.11 (The Python Software Foundation, Wilmington, DE, USA) and SPSS software version 30.0 (IBM Corp., Armonk, NY, USA), with significance set at *p* < 0.05.

## Results

The baseline characteristics of the study population are summarized in Table 1. The 93 patients had a mean age of 15.0 ± 8.7 years; 69.9% were male. Hepatic adenomas were detected in 13 patients (14%).

**Table 1:** Baseline characteristics of the study population

Characteristic	*n* = 93
Male sex	65 (69.9%)
Age (years)	15.0 ± 8.7 (range: 4–43)
LS by FibroScan (kPa)	4.77 ± 1.83
CAP by FibroScan (dB/m)	263.2 ± 52.8
LS by SWE (kPa)	5.47 ± 1.65
Aspartate aminotransferase (IU/L)	44.8 ± 48.1
Alanine aminotransferase (IU/L)	47.1 ± 53.4
Gamma-glutamyl transferase (IU/L)	39.1 ± 40.8
Cholesterol (mg/dL)	202.3 ± 54.6
Triglycerides (mg/dL)	254.8 ± 170.0
Uric acid (mg/dL)	6.09 ± 2.01
Mac-2 binding protein glycosylation isomer	0.70 ± 0.24
Adenoma present	13 (14%)

Data are presented as mean ± standard deviation or number (percentage). LS, liver stiffness; CAP, Controlled Attenuation Parameter; SWE, shear wave elastography

Table 2 presents the biochemical and imaging data according to the presence of adenomas. Patients with adenomas were significantly older than those without adenomas (24.8 vs. 13.4 years, *p* < 0.001), and had significantly higher LS as measured by FibroScan (6.58 vs. 4.47 kPa, *p* = 0.004), GGT levels (81.6 vs. 32.3 IU/L, *p* = 0.001), and total cholesterol levels (237.3 vs. 196.6 mg/dL, *p* = 0.019). LS as measured by SWE, AST levels, ALT levels, and the CAP were also higher in the adenoma group than in the no adenoma group, although these differences did not reach statistical significance.

**Table 2:** Liver parameters according to adenoma status

Parameter	No Adenoma (*n* = 80)	Adenoma (*n* = 13)	*p*-value
Age (years)	13.41 ± 7.39	24.85 ± 9.71	< 0.001
LS by FibroScan (kPa)	4.47 ± 1.43	6.58 ± 2.84	0.004
CAP by FibroScan (dB/m)	259.82 ± 49.24	284.08 ± 69.59	0.225
LS by SWE (kPa)	5.35 ± 1.56	6.23 ± 2.04	0.117
Aspartate aminotransferase (IU/L)	45.08 ± 51.16	43.46 ± 22.23	0.185
Alanine aminotransferase (IU/L)	47.05 ± 56.77	47.38 ± 25.33	0.114
Gamma-glutamyl transferase (IU/L)	34.23 ± 38.26	69.08 ± 44.70	0.001
Total cholesterol (mg/dL)	196.62 ± 51.44	237.31 ± 62.61	0.019
Triglycerides (mg/dL)	229.95 ± 147.87	418.50 ± 218.35	0.004
Uric acid (mg/dL)	5.99 ± 1.94	6.73 ± 2.37	0.318
M2BPGi	0.71 ± 0.24	0.66 ± 0.22	0.505

Data are presented as mean ± standard deviation. LS, liver stiffness; CAP, Controlled Attenuation Parameter; SWE, shear wave elastography; M2BPGi, Mac-2 binding protein glycosylation isomer

**Fig. 1:**
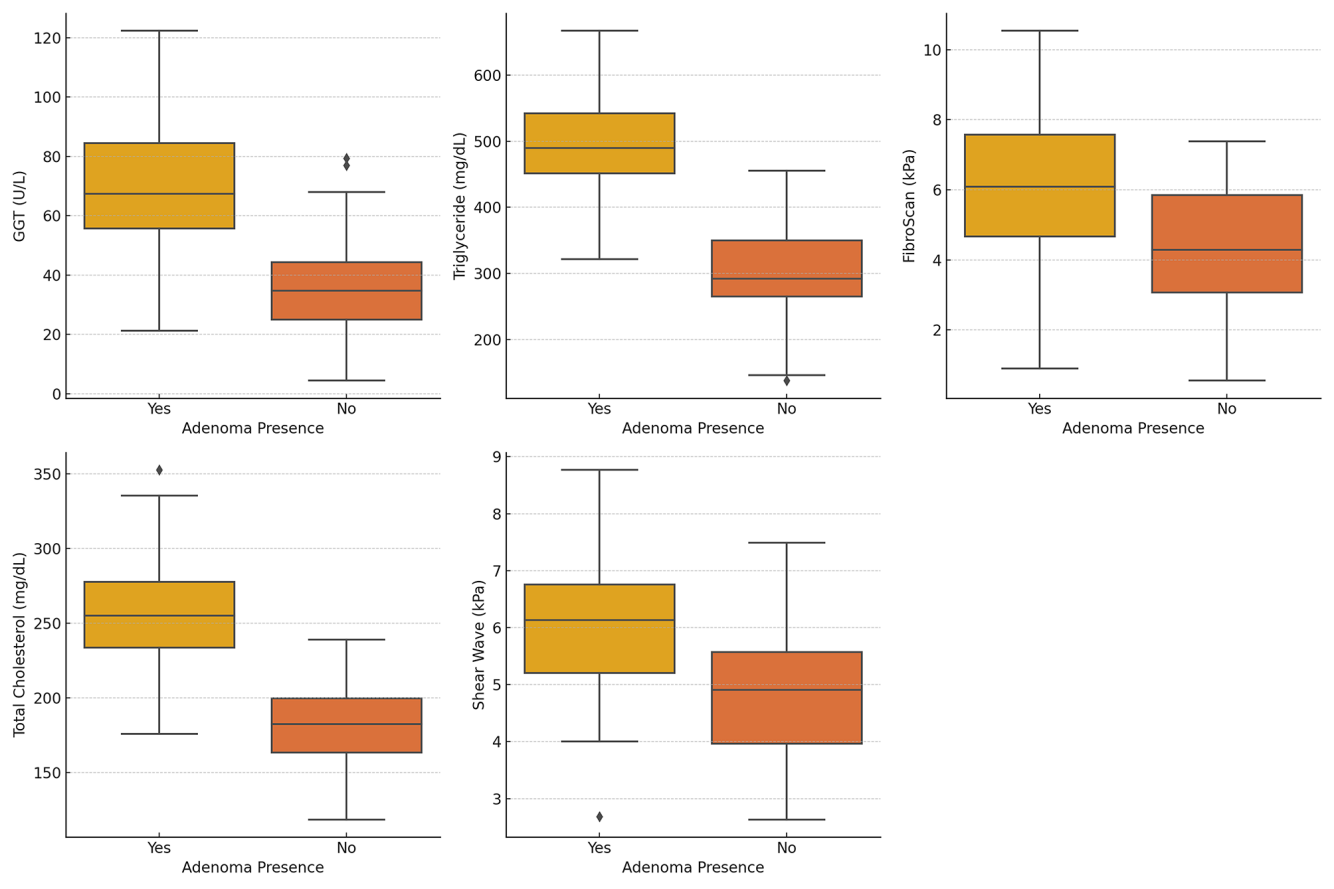
Five most discriminative liver parameters according to adenoma presence. Boxplots show GGT levels (IU/L), triglyceride levels (mg/dL), liver stiffness as measured by FibroScan (kPa), total cholesterol levels (mg/dL), and liver stiffness as measured by shear wave elastography (kPa). GGT, gamma-glutamyl transferase

Figure 1 shows the distribution of the five most discriminative features, GGT levels, triglyceride levels, LS as measured by FibroScan, total cholesterol, and LS as measured by SWE, according to adenoma status. GGT and triglyceride levels showed particularly prominent differences, consistent with their strong statistical significance (*p* = 0.001 and *p* = 0.004, respectively).

**Fig. 2:**
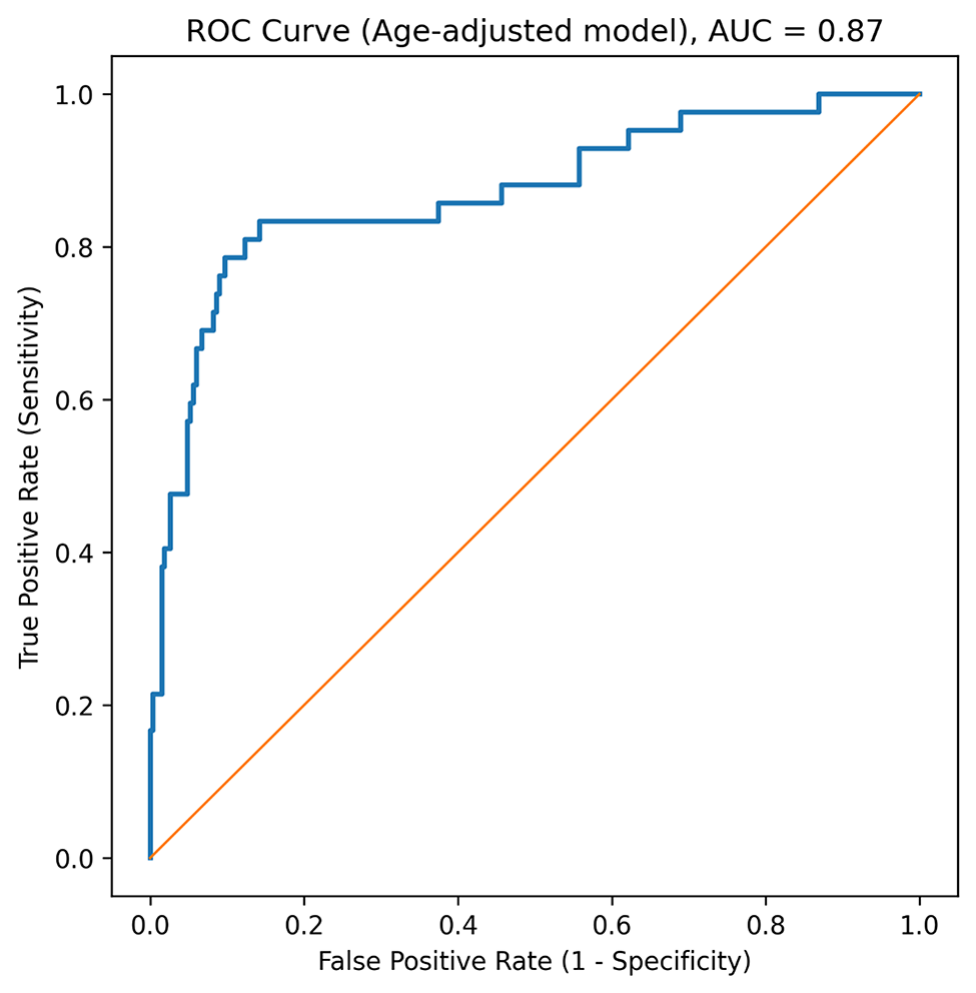
ROC curve of the adenoma prediction model. ROC curve of the logistic regression model comprised of age, gamma-glutamyl transferase level, triglyceride level, liver stiffness as measured by FibroScan, total cholesterol level, and liver stiffness as measured by shear wave elastography. The AUC of 0.87 indicated strong discriminative ability. AUC, area under the curve; ROC, receiver operating characteristic

To assess the combined predictive value of these variables, a logistic regression model was constructed. All predictor variables were entered into the regression model as raw values without standardization. The regression formula was Logit(P) = -6.41 + 0.11 × age + 0.006 × GGT + 0.004 × triglyceride + 0.28 × LS by FibroScan + 0.002 × cholesterol – 0.20 × LS by SWE where P represents the predicted probability of the presence of hepatic adenoma. The model demonstrated good discriminatory performance, with an area under the curve of 0.87 (95% confidence interval: 0.80–0.94), indicating a robust ability to distinguish hepatic adenoma despite the limited sample size (Fig. 2).

The subgroup of patients with serial imaging data showing changes in adenoma size was further analyzed to evaluate accompanying changes in clinical parameters. As summarized in Table 3, GGT levels decreased in all three groups, with a more pronounced reduction observed in patients whose adenomas decreased in size. CAP values showed slight increases in the adenoma-increased and adenoma-decreased groups and a slight decrease in the stable group.

Total cholesterol levels increased in patients whose adenomas increased in size but decreased in patients whose adenomas decreased in size or remained stable. Changes in ALT and AST levels were modest and showed no consistent directional pattern according to adenoma size dynamics. LS as measured by FibroScan decreased slightly in patients whose adenomas increased in size but increased in those whose adenomas decreased in size, while LS as measured by SWE showed small increases in both the increased and decreased groups. These findings suggest that biochemical markers, particularly GGT, may reflect dynamic changes in adenoma behavior more sensitively than elastographic parameters, which showed limited and inconsistent responsiveness to short-term size changes.

**Table 3:** Mean changes in biochemical and elastographic parameters according to changes in adenoma size

Parameter	Adenoma Increased in Size (*n* = 9)	Adenoma Decreased in Size (*n* = 11)	Adenoma Stable in Size (*n* = 23)
GGT (IU/L)	-10.00	-19.09	-3.78
CAP by FibroScan (dB/m)	+ 3.22	+ 6.09	-0.83
Triglycerides (mg/dL)	-42.14	-5.60	-11.82
Total cholesterol (mg/dL)	+ 33.11	-7.09	-12.70
LS by FibroScan (kPa)	-0.66	+ 0.82	+ 0.19
LS by SWE (kPa)	+ 0.48	+ 0.43	-0.16
ALT (IU/L)	-5.11	-4.64	-0.26
AST (IU/L)	-6.67	-1.55	+ 1.22

Data are presented as the mean change. GGT, gamma-glutamyl transferase; CAP, Controlled Attenuation Parameter; LS, liver stiffness; SWE, shear wave elastography; ALT, alanine aminotransferase; AST, aspartate aminotransferase

Table 4 summarizes the simplified threshold values for the key biochemical and imaging markers that were significantly associated with hepatic adenoma in this study; in cases where values are above the cut-off, the risk of adenoma presence or progression becomes notably higher. The cut-off values were derived from group comparisons and the logistic regression model. Cut-off values were proposed based on the mean values of significantly different variables (*p* < 0.05) between the adenoma and no adenoma groups and were rounded to a clinically meaningful threshold. These values are intended as clinical suggestions rather than established diagnostic criteria, and may aid in the early identification and monitoring of high-risk patients.

**Table 4:** Suggested clinical Cut-off values to determine adenoma risk in patients with glycogen storage disease

Parameter	Suggested Cut-off Value	Clinical Interpretation
Gamma-glutamyl transferase (IU/L)	> 60	Increased risk of adenoma; consider liver ultrasound or CAP follow-up
Triglycerides (mg/dL)	> 300	Metabolic risk likely; associated with adenoma presence
LS by FibroScan (kPa)	> 6.0	May indicate adenoma or fibrosis
CAP by FibroScan (dB/m)	> 280	Suggests hepatic steatosis; associated with adenoma progression
Total cholesterol (mg/dL)	> 220	Hypercholesterolemia; possible metabolic contributor to adenoma

LS, liver stiffness; CAP, Controlled Attenuation Parameter

## Discussion

Hepatic adenoma is a well-recognized long-term complication of GSD, particularly types I and III, with the risk increasing with age and metabolic instability. Previous longitudinal studies, such as those by Lee et al. and Rake et al., have emphasized the association between hepatic adenoma and poor metabolic control, particularly hyperlipidemia and elevated lactate, in patients with GSD [4, 11, 12]. However, these studies focused primarily on long-term outcomes and genotype–phenotype correlations, often using descriptive statistics and lacking standardized imaging tools.

In contrast, the present study used quantitative imaging (FibroScan and SWE) and structured biochemical profiling to explore both the presence and dynamic behavior of adenomas. Age, GGT levels, triglyceride levels, LS as measured by FibroScan, and total cholesterol levels were significantly associated with the presence of adenomas. A model combining these markers yielded high predictive accuracy and is clinically applicable even in resource-limited settings.

One key difference between the present and previous studies is our emphasis on the real-time assessment of progression. Whereas most previous reports, including that by Kishnani et al., have emphasized long-term risk stratification, we demonstrated that changes in GGT levels and the CAP strongly correlate with concomitant changes in adenoma size [5]. This is consistent with the inflammatory and steatotic nature of adenoma, which may result in earlier changes in metabolic markers than in LS.

Interestingly, despite their value in detecting liver fibrosis and stiffness, FibroScan and SWE did not strongly correlate with changes in adenoma size [13]. This suggests that while these imaging modalities are useful for adenoma detection, they may not reflect short-term tumor activity as sensitively as biochemical markers. Another novel insight from our study is the role of triglycerides, which showed significant differences in the cross-sectional analysis but limited longitudinal correlation. This suggests that hypertriglyceridemia may be a predictor of adenoma formation but not progression.

Taken together, our results emphasize that biochemical inflammation, as indicated by GGT levels, and hepatic steatosis, as indicated by the CAP, may predict or reflect hepatic adenoma growth more closely than LS metrics. Thus, these parameters should be considered not only diagnostic aids but also early warning indicators in the surveillance of patients with GSD. Although M2BPGi is a sensitive marker of liver fibrosis and disease activity in various chronic liver diseases including hepatitis and nonalcoholic steatohepatitis, it did not show a meaningful association with the presence or progression of hepatic adenoma in patients with GSD in the present study [14, 15]. Hepatic adenomas in patients with GSD are likely to be influenced more by metabolic and inflammatory triggers than by fibrosis alone, which may explain the lack of an association with M2BPGi levels. Therefore, although M2BPGi remains useful for broader liver disease evaluation, it may have limited utility in hepatic neoplasia surveillance in patients with GSD.

FibroScan is a reliable noninvasive liver assessment tool, the use of which is recommended by current clinical practice guidelines for metabolic dysfunction-associated steatohepatitis [16–18]. It has been widely adopted as a method of monitoring liver disease owing to its ability to measure LS and fat accumulation without the need for biopsy. Our findings suggest its additional utility in patients with GSD, as FibroScan parameters correlated with the presence of hepatic adenoma and may serve as valuable markers for the noninvasive surveillance of these patients. This finding supports the broader application of FibroScan as a practical and patient-friendly modality for liver monitoring in diverse metabolic liver diseases. We propose that in clinical practice, routine monitoring of GGT levels and the CAP could allow the timely detection of adenoma growth, enabling earlier intervention or imaging follow-up and potentially reducing the risk of hemorrhagic or malignant transformation.

Beyond statistical modeling, we propose clinically interpretable cutoff values for key biochemical and imaging markers associated with adenoma risk to offer practical guidance in real-world settings, particularly outpatient decision-making. For example, a GGT level persistently above 60 IU/L or a CAP value exceeding 280 dB/m may warrant earlier imaging follow-up even in the absence of radiological findings. Maintaining these parameters below the determined thresholds may represent a reasonable surveillance goal in patients with stable GSD. This study bridges the gap between data and daily practice by translating statistical findings into simplified thresholds to offer interpretive guidance.

This study has several limitations. First, it is a single-center retrospective study with a limited number of adenoma cases, which may affect generalizability. The small number of adenoma events (*n* = 13) relative to the number of predictors may make the model vulnerable to overfitting; external validation is essential to confirm the robustness of the model. Second, only patients who underwent FibroScan or SWE were included, potentially introducing selection bias. Third, key variables related to metabolic control, including dietary adherence and cornstarch compliance, were not consistently documented and therefore could not be analyzed, despite their known relevance to adenoma development.

## Conclusions

This study identified routine biochemical and imaging markers that may allow the identification and monitoring of hepatic adenomas in patients with GSD. GGT levels, triglyceride levels, LS as measured by FibroScan, total cholesterol levels, and LS as measured by SWE were elevated in patients with adenoma, and the logistic model constructed using these components achieved strong predictive accuracy. Changes in GGT levels and the CAP outperformed structural stiffness measures in terms of reflecting adenoma progression, suggesting that biochemical markers provide earlier insights into adenoma activity. Incorporating the markers identified here into routine monitoring, using the simple recommended clinical thresholds, may enable earlier detection of hepatic adenoma and personalized management, ultimately improving the outcomes of high-risk patients with GSD.

## Data Availability

The datasets generated and analyzed during the current study are not publicly available due to patient confidentiality.

## Abbreviations

ALT: Alanine aminotransferase
AST: Aspartate aminotransferase
CAP: Controlled Attenuation Parameter
GGT: Gamma-glutamyl transferase
GSD: Glycogen storage disease
kPa: Kilopascal
LS: Liver stiffness
M2BPGi: Mac-2 binding protein glycosylation isomer
SWE: Shear wave elastography
